# Efficacy of oxytocin administration early after psychotrauma in preventing the development of PTSD: study protocol of a randomized controlled trial

**DOI:** 10.1186/1471-244X-14-92

**Published:** 2014-03-28

**Authors:** Jessie L Frijling, Mirjam van Zuiden, Saskia BJ Koch, Laura Nawijn, J Carel Goslings, Jan S Luitse, Tessa H Biesheuvel, Adriaan Honig, Fred C Bakker, Damiaan Denys, Dick J Veltman, Miranda Olff

**Affiliations:** 1 Department of Psychiatry, Academic Medical Center, University of Amsterdam, Meibergdreef 5, 1105 AZ Amsterdam, The Netherlands; 2 Trauma Unit, Department of Surgery, Academic Medical Center, Meibergdreef 9, 1105 AZ Amsterdam, The Netherlands; 3 Department of Surgery, VU University Medical Center, De Boelelaan 1117, 1081 HZ Amsterdam, The Netherlands; 4 Department of Psychiatry, Sint Lucas Andreas Hospital, Jan Tooropstraat 164, Amsterdam 1061 AE, The Netherlands; 5 Netherlands Institute for Neuroscience, an Institute of the Royal Netherlands Academy of Arts and Sciences, Meibergdreef 47, 1105 BA Amsterdam, The Netherlands; 6 Department of Psychiatry, VU University Medical Center, Amsterdam, De Boelelaan 1117, 1081 HZ Amsterdam, The Netherlands; 7 Arq Psychotrauma Expert Group, Nienoord 5, Diemen 1112 XE, Netherlands

**Keywords:** Post-traumatic disorder, PTSD, Early intervention, Oxytocin, Neurobiology, Randomized controlled trial, Prevention

## Abstract

**Background:**

Currently few evidence based interventions are available for the prevention of PTSD within the first weeks after trauma. Increased risk for PTSD development is associated with dysregulated fear and stress responses prior to and shortly after trauma, as well as with a lack of perceived social support early after trauma. Oxytocin is a potent regulator of these processes. Therefore, we propose that oxytocin may be important in reducing adverse consequences of trauma. The ‘BONDS’ study is conducted in order to assess the efficacy of an early intervention with intranasal oxytocin for the prevention of PTSD.

**Methods/Design:**

In this multicenter double-blind randomized placebo-controlled trial we will recruit 220 Emergency Department patients at increased risk of PTSD. Trauma-exposed patients are screened for increased PTSD risk with questionnaires assessing peri-traumatic distress and acute PTSD symptoms within 7 days after trauma. Baseline PTSD symptom severity scores and neuroendocrine and psychophysiological measures will be collected within 10 days after trauma. Participants will be randomized to 7.5 days of intranasal oxytocin (40 IU) or placebo twice a day. Follow-up measurements at 1.5, 3 and 6 months post-trauma are collected to assess PTSD symptom severity (the primary outcome measure). Other measures of symptoms of psychopathology, and neuroendocrine and psychophysiological disorders are secondary outcome measures.

**Discussion:**

We hypothesize that intranasal oxytocin administered early after trauma is an effective pharmacological strategy to prevent PTSD in individuals at increased risk, which is both safe and easily applicable. Interindividual and contextual factors that may influence the effects of oxytocin treatment will be considered in the analysis of the results.

**Trial registration:**

Netherlands Trial Registry: NTR3190.

## Background

Given the high rates of exposure to traumatic events (70-90%) [1-4] and a 7-8% lifetime prevalence of PTSD [1,5], easily applicable interventions that effectively prevent PTSD are an important public health need. Currently, little evidence is available for effective interventions that prevent the development of PTSD which can be administered early after trauma exposure [6]. Single-session psychological debriefing [7,8] as well as multiple sessions of preventive Behavioral Therapy [9] administered within 3 months following traumatic events do not reduce distress, or prevent PTSD. Recently, a pilot study found that 3 sessions of prolonged exposure therapy administered within 2 weeks after trauma reduced post-traumatic stress reactions at 1 and 3 months post-trauma [10]. Other secondary preventive psychological interventions, such as brief Cognitive Behavioral Therapy (CBT), have yielded promising results [11,12] but can be applied only several weeks after trauma, when trauma-exposed individuals may already have developed acute PTSD.

Pharmacologically, prolonged administration of the beta-receptor blocking agent propranolol early after trauma did not result in fewer PTSD symptoms [13,14]. In a small sample of participants Zohar et al. [15] recently showed that a single bolus of high dose hydrocortisone in trauma-exposed individuals at an Emergency Department (ED) resulted in fewer PTSD symptoms at 2 weeks and 3 months post-trauma relative to those who received placebo. Similarly, in another recent report of 64 traumatic injury patients it was demonstrated that those who received a 10-day course of low dose oral hydrocortisone started within 12 hours of the injury reported fewer PTSD and depression symptoms at 1 and 3 months post-trauma follow-up than those who were treated with placebo [16].

Developing interventions that target vulnerability factors associated with PTSD development is a promising way to explore new early treatment strategies for prevention [17-19] An increased risk of PTSD development is associated with pre-existing dysregulations of (para)sympathetic [17,18] and hypothalamic-pituitary-adrenal axes [20,21], as well as dysregulations of central fear responses prior to [22] and shortly after trauma exposure [23,24]. In addition, a lack of perceived social support early after trauma is strongly related to increased PTSD risk [25-27].

Intranasal administration of the neuropeptide oxytocin is a candidate preventive pharmacological intervention after trauma, since oxytocin regulates neuroendocrine, psychophysiological and fear responses as well as socio-emotional processes [28].

Oxytocin is synthesized in the hypothalamus from where it is widely distributed in the brain [29]. In addition, oxytocin is released into the bloodstream by the pituitary gland where it acts as a hormone and stimulates smooth muscle tissue contraction in e.g. childbirth and lactation. In pioneering studies on the role of oxytocin in social behavior, it was found to facilitate pair-bonding and partner preference in the socially monogamous prairie vole [30,31]. Human endogenous oxytocin levels increase during safe social contact [32,33], but also during distress. Increased oxytocin activity during distress [34,35] and its attenuating effects on HPA axis [36] and autonomic nervous system activity [37] may indicate a regulatory function of oxytocin in physiological stress.

In humans, intranasal oxytocin administration is thought to result in endogenous release of the hormone in a feed-forward fashion [38]. Indeed, the recent finding that a single intranasal administration of oxytocin (16 IU) resulted in elevated salivary oxytocin levels up to 7 hours post-administration while the peptide has a half time of approximately 10 minutes, supports this hypothesis [39].

Several human studies showed that intranasal oxytocin can facilitate trust and prosocial behavior [40,41]. Furthermore, intranasal oxytocin regulates responses of the HPA axis [42] and the (para)sympathetic nervous system [43]. In addition, intranasal oxytocin also dampened the central fear response by lowering amygdala activity [44-46] and potentially enhancing fear regulation by increasing top-down control of the prefrontal cortex over the amygdala [47].

Recently, a variety of interindividual and contextual factors that influence the effects of intranasal oxytocin have come to light. Factors such as gender [48], attachment style [49], and early parental experiences [50] appear to moderate the effects of intranasal oxytocin on several outcome measures [51,52]. These findings imply that the effects of intranasal oxytocin need to be assessed accounting for these potentially moderating factors.

Preclinical studies have provided support for oxytocin treatment as a promising strategy for preventing PTSD-like behavior. In rats, a single central oxytocin administration either immediately or 7 days after a severe stressor was associated with reduced PTSD-like behavior 1 week after administration in comparison to placebo [53]. Furthermore, central oxytocin administration in rats 10 minutes prior to fear conditioning did not affect fear conditioning, but did subsequently decrease fear expression and facilitated fear extinction [54]. The same study showed that central oxytocin administrated 10 minutes prior to extinction training (at 1 day after fear acquisition) inhibited fear extinction, indicating that timing of oxytocin administration relative to traumatic memory consolidation may be important in determining whether oxytocin promotes or inhibits fear extinction. However, this finding is not supported by the study of Cohen et al. [53], where both treatment times (i.e. immediately after severe stress exposure or 7 days later) showed a similar decrease in PTSD-like behavior.

To date, no reports on the effects of intranasal oxytocin in recently traumatized human individuals have been published. One very small study showed beneficial acute effects of intranasal oxytocin in patients who had already developed PTSD. In 18 PTSD patients a reduction in anxiety, restlessness, irritability and even acute PTSD symptoms was found 50 minutes after a single dose of oxytocin compared to placebo treatment [55].

In summary, based upon these findings and given the well-documented vulnerability factors for PTSD development, we propose that intranasal oxytocin applied early after trauma may prevent the development of PTSD, through regulating fear and stress responses and socio-emotional processes such as perceptions of social support [28].

### Research aims and hypotheses

The primary aim of the ‘BONDS’ (*Boosting Oxytocin after trauma: Neurobiology and the Development of Stress-related psychopathology*) study is to investigate the effectiveness of early intranasal oxytocin administration in reducing PTSD symptoms at 1.5 month post-trauma in trauma-exposed ED patients at increased risk of PTSD. We expect that the oxytocin group will report fewer PTSD symptoms at the follow-up assessment compared to the placebo group.

As a secondary aim we will investigate whether intranasal oxytocin affects PTSD severity scores at 3 and 6 months follow-up and other psychopathology symptoms (e.g. major depressive disorder, panic disorder, specific phobia) and quality of life at 1.5, 3 and 6 months follow-up.

We will also assess moderating effects of gender, trauma type (e.g. motor vehicle accident, assault, etc.) type, history of (childhood) trauma, coping style, attachment style, and perceived social support on the main study outcome measures. Furthermore, we will investigate differences in psychophysiological, neuroendocrine, and epigenetic measures between intervention groups at 1.5 month follow-up. We hypothesize that baseline characteristics will moderate the effects of intranasal oxytocin and that the experimental intervention will be associated with more favorable outcomes on psychological, psychophysiological, neuroendocrine and epigenetic measures at the follow-up assessments compared to placebo treatment.

## Methods/Design

The ‘BONDS’ study is supported by the Netherlands Organization for Health Research and Development (ZonMw, grant no. 91210041) and by the AMC Research Council (grant no. 110614). The study has been approved by the Institutional Review Board of the Academic Medical Center (AMC) (registration number 11/273) and is conducted following guidelines of Good Clinical Practice (GCP) in accordance with the principles of the Declaration of Helsinki. The trial has been registered in the Netherlands Trial Registry and can be found at http://www.trialregister.nl (NTR3190). To assure the quality of our study, independent quality monitoring of the trial is performed.

### Study design

**Figure 1:**
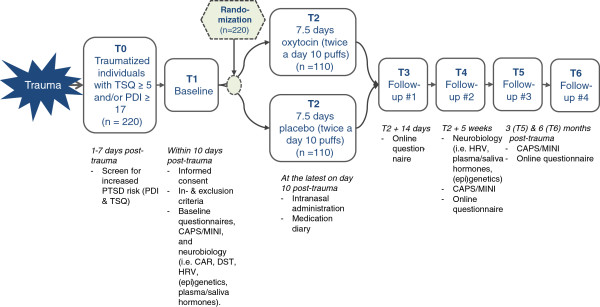
**Flowchart of the BONDS study.** CAPS Clinician Administered PTSD Scale; CAR cortisol awakening response; DST dexamethasone suppression test; HRV heart rate variability; MINI MINI International Neuropsychiatric Interview; PDI Peritraumatic Distress Inventory; TSQ Trauma Screening Questionnaire.

The ‘BONDS’ study is a multicenter double-blind randomized placebo-controlled trial in recently traumatized ED patients with increased risk of PTSD. The study design was set up with 2 baseline assessments (T1-T2) and 4 post-intervention assessments (T3-T6) (Figure 1). Participants will be randomly assigned to 1 of the 2 intervention groups, stratified by gender, ensuring that both genders are equally distributed over the 2 intervention groups.

#### Participants

We will recruit 220 patients (males and females) between 18–65 years of age who visited 1 of the 3 participating EDs in Amsterdam (of which 2 level 1 Trauma Centers) after a potentially traumatic event according to the PTSD A1 criterion in the DSM-IV (i.e. event involving actual or threatened death or serious injury, or threat to physical integrity of self or others). Only individuals who score above the cut-off on screening questionnaires indicating increased risk of PTSD development, speak Dutch or English fluently and do not meet any of the exclusion criteria (see 'List of inclusion and exclusion criteria of the BONDS study'). are eligible to participate. Furthermore, patients are also excluded when they are already randomized in a currently ongoing randomized clinical trial (RCT) conducted at the Trauma Resuscitation Room of 1 of the participating Trauma Centers (AMC) (Netherlands Trial Registry no. NTR2607).

List of inclusion and exclusion criteria of the BONDS study:

Inclusion criteria

• Presentation at the Trauma Room or Emergency Department after a potentially traumatic event, according to PTSD A1 criterion in the DSM-IV (either as a patient or direct witness)

• Trauma Screening Questionnaire (TSQ) ≥ 5 or Peritraumatic Distress Inventory (PDI) ≥ 17, preferably between 24 and 72 hours after trauma exposure, but in case of contacting difficulties up to 7 days after trauma

• Age 18 – 65 years

• Capable to read and comprehend either the Dutch or English language

Exclusion criteria

• Severe or chronic systemic disease

• Current psychotic, bipolar, substance-related, severe personality disorder, or mental retardation

• Current severe depressive disorder

• Prominent current suicidal risk or homicidal ideation

• Severe cognitive impairment or a history of organic mental disorder

• Evidence (severe) of PTSD or depression immediately prior to the index trauma

• History of neurological disorders (e.g., traumatic brain injury, seizure history)

• Reports of ongoing traumatization (e.g., in case of ongoing domestic violence)

• Evidence of clinically significant and unstable medical conditions in which OT administration is contra-indicative such as cardiovascular, gastro-intestinal, pulmonary, severe renal, endocrine or hematological disorders, glaucoma, or a stroke or myocardial infarction within the past year

• Use of certain medication: prostaglandins, certain anti-migraine medications (ergot alkaloids), β-adrenergic receptor-blocking agents, and systemic glucocorticoids.

• Sensitivity or allergy for OT or its components (e.g., methylhydroxybenzoate and propylhydroxybenzoate)

• Impaired consciousness, amnesia or confusion (objectified by Glasgow Coma Scale lower than 13 at time of inclusion)

• Female participants: pregnancy and breast-feeding (NB. Female participants with childbearing potential must have a negative pregnancy test)

#### Recruitment procedures

All ED patients are informed by posters and information brochures that it is possible that they are contacted for scientific research purposes due to their visit to the ED. In case they do not wish to be contacted, they are able to indicate this to the ED staff and/or researchers.

A member of the research team will identify potentially trauma-exposed ED patients using the ED medical records. Within 1 week after potential trauma exposure, the identified individuals are contacted by telephone or at the clinical ward to provide information about the screening procedure (T0). After verbal consent, the Trauma Screening Questionnaire (TSQ) [56] and Peritraumatic Distress Inventory (PDI) [57] are administered to assess increased risk of PTSD development. Those who score above the cut-off of the TSQ (5) [58] and/or PDI (17) [59,60] and do not meet any of the exclusion criteria receive written and verbal information about the study and are subsequently invited to participate.

#### Study procedures

After verbal and written informed consent, participants will receive a pre-intervention assessment (T1) for an extensive check of in- and exclusion criteria and to collect baseline characteristics (i.e. current PTSD symptoms, history of and current other psychopathologies, demographics, and several questionnaires assessing psychological functioning). Only those participants eligible for further participation will be randomized. At home, participants will collect saliva samples for the assessment of the cortisol awakening curve and dexamethasone suppression test on 2 consecutive days. During a second appointment (T2), baseline measures of basal psychophysiological functioning are determined. Venous blood and saliva is collected for the analysis of neuroendocrine and (epi)genetic material. Subsequently, participants receive instructions on how to use the nasal spray and will apply the first dose in the presence of the researcher. Two weeks after the start of the intervention, participants receive an online questionnaire to assess psychological functioning (T3).

One month after the end of the intervention (T4) (i.e. approximately 1.5 month post-trauma) the severity of PTSD and the presence of other psychopathology symptoms are assessed. Additionally, blood, saliva, hair, and psychophysiological measures are collected. At the remaining follow-up sessions at 3 (T5) and 6 months (T6) after the index trauma, the severity of PTSD and the presence of other psychopathology symptoms will be re-assessed and a subset of questionnaires will be re-administered.

#### Intervention

Participants will be blindly allocated to either intranasal oxytocin (Syntocinon®, 40 IU/ml, registered in the Netherlands as RVG 03716) or placebo (Sodiumchloride (NaCl) nasal spray 0.8% based on the Formulary of Dutch Pharmacists). The bottles are re-labeled so that the oxytocin and placebo bottles appear identical. All study medication is prepared by the hospital pharmacy of the Slotervaart Hospital, Amsterdam, under Good Manufacturing Practice (GMP) licence. Participants receive a total of 120 ml of nasal spray, divided over 3 bottles.

Participants are instructed to self-apply a total of 15 doses of 10 puffs of their allocated treatment, i.e. 40 IU oxytocin per dose for the oxytocin condition. A dose of 40 IU twice a day is comparable to the doses previously used in human studies investigating effects of multiple treatments of oxytocin [61,62] and does not produce significant side effects or adverse outcomes [63]. The first intranasal dose is self-applied under researcher supervision at the latest on day 10 post-trauma exposure (T2). The following 14 doses will be administered in the morning and evening of the 7 consecutive days, preferably with 12-hour time intervals. Participants are asked to keep a medication diary to register the time of each administration and any possible side effect or adverse event.

#### Screening instruments

Acute PTSD symptoms are assessed with the Trauma Screening Questionnaire (TSQ) [56] a screening instrument adapted from the PTSD symptom scale-self report [64]. The TSQ consists of 10 dichotomous items (5 re-experiencing and 5 arousal items) from the DSM-IV PTSD criteria [65] The optimal TSQ cut-off score to predict PTSD at 1 month after trauma is 6, as was found in a sample of trauma-exposed individuals who where screened at 1 to 3 weeks post-trauma [58]. Since we administer the questionnaire relatively early post-trauma (i.e. within the first week compared to 1 to 3 weeks post-trauma) and a cut-off score of 5 still has a good sensitivity (0.84) and specificity (0.92) [58], we use the lower cut-off score of 5 to signal increased risk. In addition, due to the short time period since the trauma (i.e. preferably 3 days), we ask whether the individual experienced the item at least once since the event (instead of twice). The Dutch version has a good reliability and validity [66]. In our own research group, we have observed that a score of 5 or higher on the TSQ provided optimal sensitivity and specificity for PTSD diagnosis at 1 month post-trauma in Trauma Resuscitation Room patients [60].

The extent of distress during and immediately after the event is measured with the Peritraumatic Distress Inventory (PDI) [57,67]. The PDI is an internally consistent 13-item scale. Items are answered on a 5-point scale (range: 0–52). Higher PDI scores obtained within a few days after trauma have found to predict PTSD at 1 month post-trauma in a population of motor vehicle casualties [59]. In our own research group, we have observed that a score of 17 or higher on the PDI provided optimal sensitivity and specificity for PTSD diagnosis at 1 month post-trauma in Trauma Resuscitation Room patients [60].

#### Primary outcome measure

##### Diagnostic clinical interviews

PTSD symptoms are assessed with the Clinician-Administered PTSD Scale (CAPS) [68]. The CAPS is 1 of the most widely used structured clinical interviews for diagnosing PTSD according to DSM-IV criteria (note: at the beginning of the recruitment phase, the DSM-5 had not yet been published). It measures PTSD severity using the symptom clusters re-experiencing (B-cluster, 5 symptoms), avoidance (C-cluster, 7 symptoms) and hyperarousal (D-cluster, 5 symptoms). The CAPS distinguishes between the estimated frequency (range: 0–4) and intensity (range: 0–4) of the various symptoms. Frequency and intensity scores are added up to a total CAPS score (range: 0–136). The Dutch version has been validated [69].

#### Secondary outcome measures

##### Diagnostic clinical interview

The MINI International Neuropsychiatric Interview (MINI) clinical interview [70,71] is a widely used structured clinical interview used to diagnose current and lifetime DSM-IV psychiatric disorders. It is used to assess the presence of psychopathology other than PTSD and lifetime PTSD. The Dutch version has been validated [71].

##### Self-report measures

Demographics characteristics including gender, age, social-economic status (SES), ethnic and cultural background, medication use, medical history, smoking, nicotine, alcohol and other drug use, sexual orientation, and relationship status are collected.

Current anxiety and depression symptoms (in the past week) are measured with the Hospital Anxiety and Depression Scale (HADS) [72,73], a well-established 14-item (4-point scale) questionnaire containing 2 subscales: HADS-A (Anxiety, 7 items, range: 0–21) and HADS-D (Depression, 7 items; range: 0–21). Higher scores indicate more depressive/anxious symptoms. The reliability was high in a sample of Dutch traumatized persons [73].

Self-reported PTSD symptoms in the past week are assessed using the Impact of Event Scale – Revised (IES-R) [74] consisting of 22 items (5-point scale) divided in 3 subscales corresponding to the 3 PTSD symptom clusters in the DSM-IV: re-experiencing (8 items, scale range 0–32), avoidance (8 items, range 0–32) and hyperarousal (6 items, range 0–24). Higher scores indicate more PTSD symptoms. The Dutch version has been validated [60].

Level of fatigue in the past 2 weeks is measured with the Checklist Individual Strength (CIS) [75]. The CIS uses 20 items (7-point scale) divided over 4 subscales (fatigue severity, concentration problems, reduced motivation, and reduced activity). A total fatigue score is calculated by the sum score of all items (range 20–140). The survey has a good reliability and has been well validated.

Perceived health problems are assessed using a Dutch translation of the Subjective Health Complaints inventory (SHC) [76]. The SHC consists of 29 items on subjective somatic and psychological complaints experienced during the last 30 days (4 point scale: 0 (no complaints) to 3 (severe complaints), range 0–87). The survey has been tested and has satisfactory validity and reliability [76].

Quality of Life over the past 2 weeks is measured using the brief version of the World Health Organization Quality of Life survey (WHOQOL-BREF) [77]. The questionnaire comprises 26 items, including 2 general items on quality of life and 24 items covering 4 domains: physical health, psychological health, social relationships, and environment. The questions have 5-point Likert scales. The Dutch WHO-QOL-BREF has good validity and reliability [78].

To assess the experience of happiness, pain, and sexual functioning currently and over the past month, 6 questions ask for subjective feelings regarding these items on a 10-point scale, each point accompanied by a corresponding smiley face.

During the psychophysiological and neuroendocrine assessments subjective rates of acute stress are assessed with the State and Trait Anxiety Inventory-state version (STAI-state) [79] The STAI-state consists of 20 4-point scale items (range 20–80) and the Dutch version has been validated [80]. Subjective feelings of tension, anxiety, happiness and calmness are assessed with visual analogue scales (VAS, scales 0–100).

#### Self-report measures for exploratory subgroup analyses

These measures will additionally be used for exploratory subgroup analyses of the effects of the intervention.

General subjective feelings of social support are measured with the short version of the Social Support List (SSL-6) [81], consisting of 6 items (4-point scale) about the amount of different types of support one receives (range 6–24). Additionally, 6 items assess the satisfaction with the amount of received support (range 6–24). The list has good construct validity and high reliability [81].

Attachment style is measured using the Experiences in Close Relationships Scale (ECR) [82] which dimensionally measures attachment anxiety (18 items) and attachment avoidance (18 items) (7-point Likert scale). Scores range between 18–49 for both subscales, with higher scores indicating a more anxious or avoidant attachment style. The questionnaire has a high reliability and validity [83].

Coping strategy is measured with the Dutch Brief Coping Strategy Indicator (DUBRISCI), a brief version of the Coping Strategies Indicator (CSI) [84]. The questionnaire consists of 9 items (3-point scale), to assess 3 basic modes of coping: problem solving, seeking social support, or avoiding the event (range 0–18). The CSI has been found to be valid and reliable [84].

Peritraumatic dissociation is assessed with the Peritraumatic Dissociation Experience Questionnaire (PDEQ) [85] and uses 10 items of peritraumatic dissociative reactions to a traumatic event on a 5-point scale (range 10–50). The PDEQ is valid and reliable [86].

History of potential childhood trauma is assessed with the short version of the Early Trauma Inventory-Self report (ETI) [87]. The 27-item questionnaire assesses the number and frequency of different types of potentially traumatic experiences (physical, sexual, and emotional abuse and general traumas) and has been shown to be a valid and reliable measure of early trauma.

### Biological measures - Psychophysiology

Heart rate (HR) and heart rate variability (HRV) are assessed with the Polar RS800CX (wrist watch and chest strap). R-R intervals obtained from the equipment will be used to calculate high-frequency HRV (HF-HRV) as an index for parasympathetic nervous system functioning [88].

### Biological measures - blood and saliva

#### Neuroendocrine measures

DHEAS and cortisol are HPA-axis indices and can be determined in saliva. We will use Salivettes (Salivettes, Sarstedt, Rommelsdorf, Germany) for the assessment of DHEAS and cortisol at T2 and T4 (under resting conditions). In addition, to assess normal circadian cortisol levels and cortisol suppression by dexamethasone (DEX) from saliva, participants are instructed to collect 5 saliva samples per day on 2 consecutive days prior to the start of the intervention (time points: at awakening, +15, +30, and +60 minutes after awakening and in the evening before going to bed). Basal cortisol levels are assessed during the first day of saliva collection. A low dose (0.25 mg) of dexamethasone (DEX) is administered at 11:00 pm during the first day [89], to measure cortisol levels after suppression with DEX on the second day.

To assess retrospective cortisol levels, cortisol levels are measured in hair [90]. Fifteen mg of hair is collected at T4. Focus will be on the first 2 1 cm segments of scalp-nearest samples, representing the month prior to trauma, the month after the intervention, and a time period in between.

Saliva samples will be stored at -20°C and hair samples at room temperature until further analysis.

Oxytocin and AVP concentrations will be measured in plasma. Blood samples are collected in 2 ice-chilled 6 ml EDTA tubes, placed on ice immediately after sampling, centrifuged at 4°C within 60 minutes after blood collection and stored until further analysis. Extracted plasma will be stored at -80°C until further analysis.

### (Epi)genetics

To investigate possible effects of genetic variation on the effectiveness of oxytocin treatment, we will assess carrier status of single-nucleotide polymorphisms (SNPs) which have previously been related to PTSD symptoms and/or dysregulated biological systems in PTSD (e.g. OXTR, glucocorticoid receptor, FKBP5). Furthermore, DNA methylation of CPG-islands of these genes (i.e. epigenetics) will be assessed. Through DNA methylation, environmental experiences regulate gene expression, which may induce lasting changes in stress reactivity [91,92]. Differential levels of DNA methylation of genes related to stress reactivity have already been associated with PTSD and early life stress [93-95]. These measures will be used for exploratory subgroup analyses of the effects of the intervention. For the collection of (epi)genetic material 6 ml of whole blood is collected in an EDTA tube, which is kept at 7°C until DNA is extracted and stored by a certified laboratory.

#### Statistical analysis - primary outcome

To assess the efficacy of the oxytocin intervention, we will analyze the difference in mean CAPS scores between the 2 arms of the trial at 1.5 month post-trauma (T4). Descriptive statistics will be used for exploration of the data. All analyses will be conducted on the basis of intention-to-treat (ITT). Results will be expressed as differences in mean scores between the 2 groups with 95% confidence intervals**.** P-values <0.05 will be considered to indicate statistical significance.

#### Statistical analysis - secondary outcomes and exploratory analyses

Additionally, we will analyze the difference in mean CAPS scores between the 2 arms of the trial at 3 (T5) and 6 (T6) months post-trauma. We will investigate the difference in levels of depression and other psychopathology symptoms between the 2 trial arms during the follow-up assessments. Changes in continuous symptom and physiological and neuroendocrine measures between groups from pre- to post-intervention will be evaluated by longitudinal analyses using linear mixed models. In multivariate analyses we will investigate potential confounding factors, such as gender and age. Based on earlier studies on early interventions to prevent PTSD [12] and on different interindividual responses to intranasal oxytocin administration [51,52] relevant exploratory subgroup comparisons will be made to examine whether treatment effects differ between specific subgroups. We will analyze if variables such as subjective social support, attachment, coping style, (epi)genetics, peritraumatic dissociation, and history of childhood trauma, moderate the effect of the intervention on our outcome measures.

#### Sample size calculation

Sample size calculation was based on the minimal effect size considered to be clinically relevant in the prevention of PTSD symptom development and on previous results of studies on the effects of long-term oxytocin administration in reducing psychiatric symptoms. We used the program NQuery Advisor® 7.0 [96] to calculate minimal group sizes. A previous study in a similar population by our department has shown that we can expect a standard deviation between 19.93-23.81 points on the CAPS score at approximately 1 month after trauma [60]. Based on a SD of 23.81, a small-to-medium effect size of d = 0.4 would result in a 9.52-point lower CAPS score in the oxytocin-treated group compared to the placebo group at 1 month post-intervention (a CAPS score of 45 is required for a PTSD-diagnosis). We consider this group difference on the CAPS to be clinically relevant.

Support for the notion that an effect size of d = 0.4 adequately reflects the potential of oxytocin to reduce psychiatric symptoms stems from 2 previous studies on long-term intranasal oxytocin administration in the treatment of positive and negative symptoms in schizophrenia patients [61,97]. The observed effect sizes were between d = 0.05 - 0.88 [97] and d = 0.24 -.74 (for most outcomes around d = 0.4) [61], depending on the outcome measure. Noteworthy, oxytocin treatment greatly reduced anxiety symptoms in schizophrenic patients (d = 0.62) [97].

To be able to detect a group difference with an effect size of d = 0.4 using a two sample t-test with an alpha of 0.05 two-sided significance level and power of 80%, 100 participants per trial arm are needed. To allow for 10% attrition (estimated from our previous study in a similar population) we will include 110 participants in each study group at T1. Therefore we will include 220 participants in total.

#### Safety and monitoring procedures of (serious) adverse events

All adverse events reported spontaneously by the participant or observed by the research team will be recorded. All adverse events will be judged on intensity and its relation with the investigational product. Adverse events will be followed until they have abated, or until a stable situation has been reached.

All serious adverse events (SAEs; include events that are life-threatening, require hospitalization, result in persistent or significant disability or incapacity, are a congenital anomaly or birth defect, or is a new event of the trial likely to affect the safety of the participants) will be reported to the IRB of the AMC and the competent authority of the Netherlands, within 15 days after the research team has first knowledge of the SAE.

SAEs that result in death or are life-threatening are reported expedited. The expedited reporting will occur not later than 7 days after the responsible investigator has first knowledge of the adverse reaction. This is for a preliminary report with another 8 days for completion of the report. If an SAE is to occur during the nasal spray administration period, the participant will be withdrawn from treatment.

## Discussion

The ‘BONDS’ study aims to investigate the efficacy of intranasal oxytocin administration in preventing PTSD in trauma-exposed ED patients with increased risk of PTSD. This is highly relevant since evidence-based interventions that prevent PTSD in the first few weeks post-trauma are much needed. Intranasal oxytocin is a promising pharmacological agent for PTSD prevention, since it acts on risk factors associated with PTSD development, i.e. a lack of social support and dysregulated stress and fear responses. Based on previous early intervention studies, recruiting ED patients for research purposes early after trauma exposures is feasible [10,15,16,60,98].

### Strengths and limitations

A strength of the current trial is that we assess a wide range of variables that may moderate the effect of the OT intervention [54]. We measure a variety of relevant contextual and interindividual factors (e.g. gender, attachment style, coping and subjective social support), since it has recently been shown that effects of oxytocin may depend on these contextual and interindividual differences [51,52]. These factors will be considered in the analysis of the results of the ‘BONDS’ study, which allows us to determine if the intervention has diverging effects in subgroups of participants.

In prospective studies on the development of PTSD following a traumatic event, study populations commonly consist of ED patients with traumatic injuries that require hospital admission [99], since events that cause such injuries immediately meet the A1 criterion of the DSM-IV for PTSD. However, we opted for a broader recruitment strategy in order to additionally identify trauma-exposed individuals with minor injuries. Although we do exclude some trauma types (e.g. domestic violence and other ongoing traumatizing events) we believe that our more heterogeneous population will yield better generalizable results with stronger implications for general practice. In addition, it has been reported that higher objective injury severity scores due to a potential traumatic event do not predict later PTSD symptoms [100].

However, due to the acute nature of the trial and the stringent inclusion criterion of presence of increased PTSD risk, recruiting a large of number of participants is a challenging task. It proves to be difficult to contact all trauma-exposed individuals in time. However, since the BONDS study is an RCT on the efficacy of an intervention and not an epidemiological study, it remains possible to assess the potential effect of the intervention in the population that we are able to include in the study.

Another possible limitation of the trial is that there is no direct evidence that the timing we chose for the start and duration of the intervention is the most effective. As discussed above, preclinical studies differ in whether the timing of the oxytocin intervention in relation to severe stress exposure either does not influence the outcome [53] or determines whether the intervention results in beneficial or adverse effects [54]. In addition, starting intranasal oxytocin treatments at the latest at day 10 post-trauma is considerably later than the timing of the only pharmacological intervention that has currently yielded promising preventive effects, i.e. hydrocortisone within the first 12 hours post-trauma [15,16]. Furthermore, it may be hypothesized that intranasal oxytocin needs to be applied prior to (i.e. not after) trauma exposure in order to ameliorate adverse effects of traumatic stress. However, this method may only be implemented in high-risk populations, and requires caution and further research, since long-term effects of multiple oxytocin treatments over an extended time period have not been widely studied yet. In addition, the current notion is that only individuals with increased risk of PTSD should receive any form of (primary or secondary) preventive interventions [101]. Even though pre-trauma risk factors have been established [102], this knowledge cannot yet be used to assess pre-trauma individual PTSD risk.

Another possible limitation of the trial is that we do not know which dose of intranasal oxytocin is most effective, since there is a lack of dose–response studies of intranasal oxytocin. Only a few studies examined the differential effects of higher (24, 40 or 48 IU) and lower (10, 20 or 24 IU, respectively) oxytocin doses [103-105]. In these studies the dose around 20 IU (24 IU or 20 IU) had more favorable effects compared to the other dose studied (48, 40 or 10 IU). However, these studies were single administration studies on non-clinical outcome measures. We based our dose of 40 IU on 2 studies that examined the effects of prolonged intranasal oxytocin administration on clinical symptoms in (psychiatric) patient populations and observed favorable outcomes [61,62]. Another limitation with regard to the dose and administration method used is that it carries a risk of ineffective delivery, since we apply multiple sprays per dose [106].

In all, the ‘BONDS’ study is an RCT studying the preventive actions of intranasal oxytocin, a promising novel early intervention aimed at modifiable risk factors of PTSD development, in individuals exposed to trauma at increased risk of PTSD. If effective, oxytocin administration will be an easily applicable method to reduce adverse psychological outcome as a result of trauma exposure.

## Competing interests

The authors declare that they have no competing interests.

## Authors’ contributions

MO designed the study. JLF and MvZ drafted the manuscript. All authors contributed to the development and implementation of the study protocol at the Emergency Departments (Sint Lucas Andreas Hospital, AMC and VUmc) and Trauma Centers (AMC and VUmc). MO and MvZ arranged collaborations with the participating laboratories. JLF, LN, and SBJK conduct all participant-related study procedures. All authors contributed to editing the manuscripts and read and approved the final manuscript.

## Pre-publication history

The pre-publication history for this paper can be accessed here:

http://www.biomedcentral.com/1471-244X/14/92/prepub
